# Ameliorative Effects of Oyster Protein Hydrolysates on Cadmium-Induced Hepatic Injury in Mice

**DOI:** 10.3390/md20120758

**Published:** 2022-11-30

**Authors:** Jingwen Wang, Zhijia Fang, Yongbin Li, Lijun Sun, Ying Liu, Qi Deng, Saiyi Zhong

**Affiliations:** 1 College of Food Science and Technology, Guangdong Ocean University, Zhanjiang 524088, China; 2 Guangdong Provincial Key Laboratory of Aquatic Product Processing and Safety, Zhanjiang 524088, China; 3 Guangdong Provincial Engineering Technology Research Center of Marine Food, Zhanjiang 524088, China; 4 Key Laboratory of Advanced Processing of Aquatic Products of Guangdong Higher Education Institution, Zhanjiang 524088, China

**Keywords:** cadmium, oyster protein hydrolysate, hepatic injury, oxidative stress, inflammatory response, hepatocyte apoptosis

## Abstract

Cadmium (Cd) is a widespread environmental toxicant that can cause severe hepatic injury. Oyster protein hydrolysates (OPs) have potential effects on preventing liver disease. In this study, thirty mice were randomly divided into five groups: the control, Cd, Cd + ethylenediaminetetraacetic acid (EDTA, 100 mg/kg), and low/high dose of OPs-treatment groups (100 mg/kg or 300 mg/kg). After continuous administration for 7 days, the ameliorative effect of OPs on Cd-induced acute hepatic injury in Cd-exposed mice was assessed. The results showed that OPs significantly improved the liver function profiles (serum ALT, AST, LDH, and ALP) in Cd-exposed mice. Histopathological analysis showed that OPs decreased apoptotic bodies, hemorrhage, lymphocyte accumulation, and inflammatory cell infiltration around central veins. OPs significantly retained the activities of SOD, CAT, and GSH-Px, and decreased the elevated hepatic MDA content in Cd-exposed mice. In addition, OPs exhibited a reductive effect on the inflammatory responses (IL-1β, IL-6, and TNF-α) and inhibitory effects on the expression of inflammation-related proteins (MIP-2 and COX-2) and the ERK/NF-κB signaling pathway. OPs suppressed the development of hepatocyte apoptosis (Bax, caspase-3, and Blc-2) and the activation of the PI3K/AKT signaling pathway in Cd-exposed mice. In conclusion, OPs ameliorated the Cd-induced hepatic injury by inhibiting oxidative damage and inflammatory responses, as well as the development of hepatocyte apoptosis via regulating the ERK/NF-κB and PI3K/AKT-related signaling pathways.

## 1. Introduction

Cadmium (Cd) is a widespread environmental toxicant that poses a serious threat to human health [1]. Due to its high solubility in water, Cd can easily enter the human body through the food chain from polluted soils and water [2]. Cd exposure will cause metabolic dysfunction and eventually lead to irreversible damage to multiple organs [3,4]. About 50–70% of the absorbed heavy metal accumulates in the kidney and liver [5]. Acute Cd exposure primarily results in liver accumulation and hepatic injury [6]. The liver has been considered one of the main target organs of Cd [7]. Recent research has shown that acute Cd exposure leads to severe hepatic injury, accompanied by oxidative damage, inflammation, and apoptosis [8]. Therefore, reducing oxidative damage, ameliorating inflammatory response, and preventing the development of hepatocyte apoptosis may be practical strategies for the treatment of Cd-induced hepatic injury.

Protein hydrolysates from oysters (*Crassostrea hongkongensis*) have multiple health benefits, including anti-oxidation [9], anti-inflammatory [10], anti-apoptosis [11], anti-cancer, and other properties [12]. In previous studies, oyster-derived hydrolysates have been shown to be protective against D-galactosamine(D-GalN)-induced hepatic injury [13]. The peptides (SCAP1, SCAP3, and SCAP7) produced from oyster protein hydrolysis (OPs) present strong antioxidant and anti-cancer properties [14]. In addition, OPs are considered to be a safe and effective dietetic treatment for alcoholic liver disease by declining ethanol-induced oxidative stress and inflammation [15]. In addition, oyster ferritin was found to efficiently reduce the damage of heavy metals in mice [16]. The evidence suggests that OPs might be a potential candidate for ameliorating Cd-induced hepatic injury. Therefore, this study aimed to investigate the ameliorative effect of OPs on hepatic oxidative damage, inflammation, and apoptosis in Cd-exposed mice.

## 2. Results

### 2.1. Sequence Analysis of the Main Peptides of OPs

**Figure 1:**
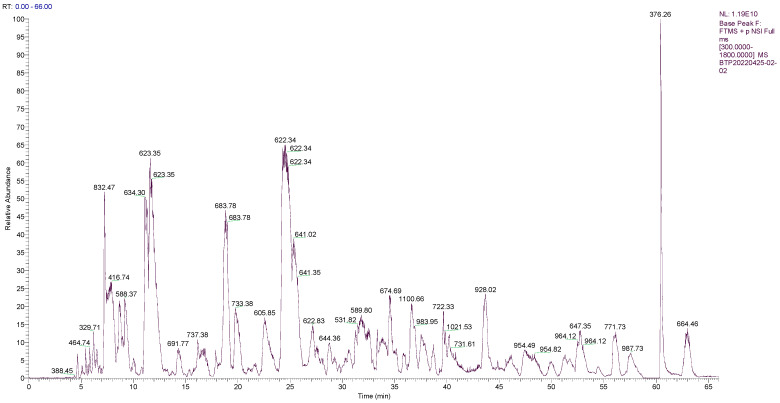
Total ion chromatogram of peptides from oyster enzymatic hydrolysates (OPs).

As shown in Figure 1, the peaks of OPs were mostly in the range of 300 to 900 *m*/*z*. Overall, 177 peptides, with molecular weights ranging from 550.250 to 1387.697 Da, and an intensity ranging from 3,857,400 to 171,200,000, were identified from the OPs. The peptide fingerprinting of 40 characteristic peptides in OPs was analyzed using liquid chromatography–tandem mass spectrometry (LC-MS/MS). The scores for evaluating the matches between the theoretical and experimental mass spectrums were obtained by comparing the UniProt database; 20 peptide sequences with higher scores are listed in Table 1. Of interest, they contain a higher percentage of hydrophobic amino acids, such as proline (P, 37/180 residues in 20 peptides), valine (V, 14/180), and alanine (A, 8/180). Specific amino acid motifs, such as PVX, was repeated six times, and PxxP was repeated eight times, X being either a glycine, serine, or a proline residue, x being either an alanine, glycine, valine, threonine, asparagine, leucine, glutamic acid, aspartic acid, or an arginine, can be recognized. Hydrophobic amino acids proline or proline-rich peptides were reported to possess good anti-Cd, anti-oxidation, and anti-inflammatory properties [17,18,19,20,21,22].

### 2.2. Composition of Amino Acid in OPs

According to the data from the automatic analyzer, the content of total amino acids in OPs was 33.73 g/100 g (Table 2). The content of essential amino acids in OPs was 11.82 g/100 g and accounted for 35.04% of the total amino acids. The content of hydrophobic amino acids was 12.76 g/100 g and accounted for 37.83% of the total amino acids. OPs were rich in Glutamic acid (Glu, 4.33 g/100 g), Aspartic acid (Asp, 3.36 g/100 g), Alanine (Ala, 2.63 g/100 g), Proline (Pro, 2.61 g/100 g), and Lysine (Lys, 2.61 g/100 g).

### 2.3. Contents of Free Amino Acids in OPs

Table 3 shows the free amino acid content and composition of OPs. The contents of total free amino acids in OPs were 15.80 g/100 g, indicating OPs contained abundant free amino acids. Among these free amino acids, Pro (2.49 g/100 g), Glu (2.12 g/100 g), Tyr (1.51 g/100 g), Lys (1.43 g/100 g), Gly (1.38 g/100 g), and Val (0.91 g/100 g) were highly detected in OPs. The essential free amino acids accounted for 33.73% of the free total amino acids, and free hydrophobic amino acids accounted for 38.86%. 

### 2.4. Effects of OPs on Hepatic Dysfunction in Cd-Exposed Mice 

**Figure 2:**
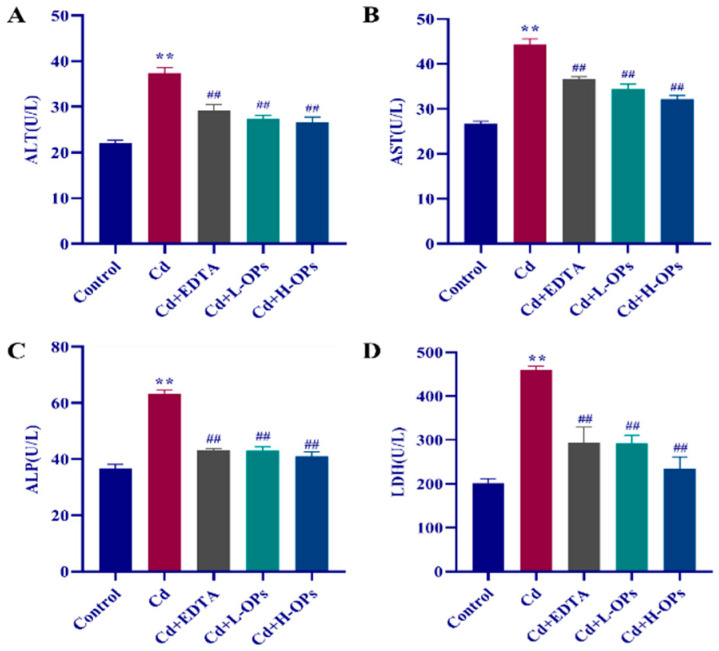
Effect of OPs on the liver function profiles in mice (**A**) ALT: Alanine aminotransferase; (**B**) AST: Aspartate aminotransferase; (**C**) ALP: Alkaline phosphatase; (**D**) LDH: Lactate dehydrogenase; Control: Intraperitoneal injection of 0.9% NaCl (saline) once daily; Cd: Mice were injected intraperitoneally with CdCl_2_ 5 mg/kg daily; EDTA: Mice were injected with CdCl_2_ (5 mg/kg) intraperitoneally after 1 h of oral administration with EDTA (100 mg/kg) daily; Cd+L-OPs: Mice were injected with CdCl_2_ (5 mg/kg) intra-peritoneally after 1 h of oral administration with a low dose of OPs (100 mg/kg) daily. Mice were injected with CdCl_2_ (5 mg/kg) intra-peritoneally after 1 h of oral administration with a high dose of OPs (300 mg/kg) daily. The data were expressed as mean ± SEM, *n* = 6 in each group. Compared with the control group, ** *p* < 0.01; compared with the Cd group, ^##^ *p* < 0.01.

Compared with the control group, the Cd-exposed mice group showed the highest levels of serum alanine transaminase (ALT), aspartate aminotransferase (AST), alkaline phosphatase (ALP), and lactate dehydrogenase (LDH) (Figure 2). EDTA therapy is the most widely used for treating patients with acute or chronic Cd disease [23]. Thus, it was used as a positive control in this test. The supplement of OPs significantly decreased the levels of serum ALT, AST, ALP, and LDH (*p* < 0.01). The effects of OPs were met even better than that of the positive agent EDTA treatment. OPs exhibited a good ameliorative effect on hepatic dysfunction in Cd-exposed mice.

### 2.5. Effect of OPs on Hepatic Injury in Cd-Exposed Mice

**Figure 3:**
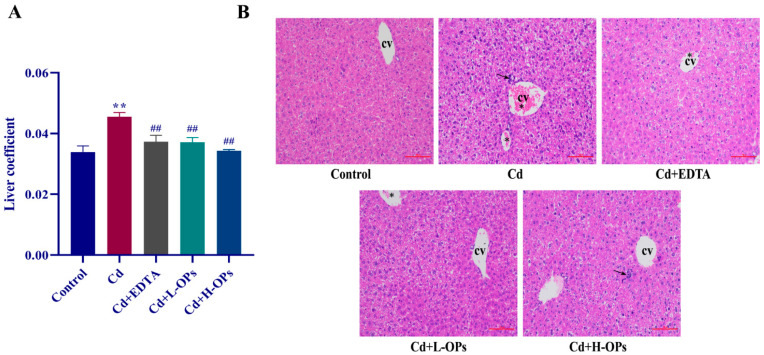
Effects of OPs on liver coefficient and hepatic injury in Cd-exposed mice. (**A**) Liver coefficient = liver weight(g)/mouse weight(g); (**B**) Histopathology with H&E staining (200×) of the liver in mice after treatment for 7 days; CV: Central veins; Arrow: lymphocyte accumulation in the parenchyma; asterisk (*): hemorrhage. Bar = 100 μm. The data were expressed as mean ± SEM, *n* = 6 in each group. Compared with the control group, ** *p* < 0.01; compared with the Cd group, ^##^ *p* < 0.01.

The organ weight coefficient is commonly used to evaluate the toxic effect [24]. The liver weight coefficient of the mice in the Cd-exposed group was significantly higher than that of the mice in the control group (*p* < 0.01). The OPs markedly lowered the liver coefficients in Cd-exposed mice (*p* < 0.01). Histopathological sections of the liver stained with H&E are shown in Figure 3B. Compared with the control group, the mice in the Cd group showed obvious pathological changes in liver tissue, including apoptotic bodies, hemorrhage, lymphocyte accumulation, and inflammatory cell infiltration around the central vein. In the OPs and EDTA-treated groups, liver tissue retained its normal appearance and had fewer apoptotic bodies. OPs showed a protective effect on liver tissue against Cd.

### 2.6. Effect of OPs on Hepatic Oxidative Indexes in Cd-Exposed Mice

**Figure 4:**
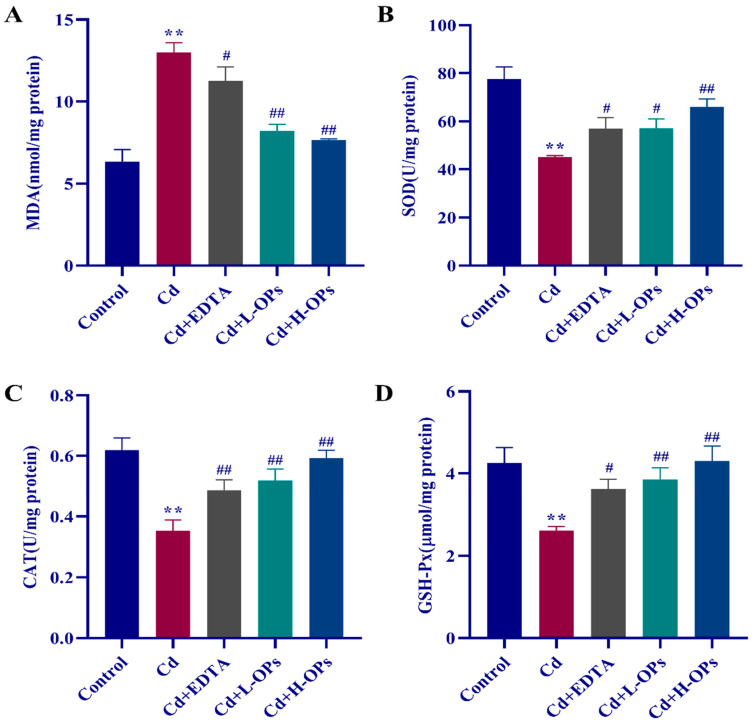
Effects of OPs on MDA level, SOD, CAT, and GSH-Px activities in Cd-induced mice. (**A**) Malondialdehyde (MDA); (**B**) Superoxide dismutase (SOD); (**C**) Catalase (CAT); (**D**) Glutathione peroxidase (GSH-Px). The data were expressed as mean ± SEM, *n* = 6 in each group. Compared with the control group, ** *p* < 0.01; compared with the Cd group, ^#^ *p* < 0.05, ^##^ *p* < 0.01.

An increased MDA level is an important indicator of oxidative stress. The Cd-exposed mice group showed the highest levels of hepatic MDA, while the OPs treatment significantly lowered the level of MDA (*p* < 0.01, Figure 4A). More importantly, the effects of OPs were better than that of EDTA. OPs markedly inhibited lipid peroxidation (MDA as an indicator) and MDA production in Cd-exposed mice. As shown in Figure 4B–D, antioxidant markers such as SOD, CAT, and GSH-Px were significantly reduced in the Cd-exposed mice group compared to the control group. OPs retained higher activity of antioxidant enzymes in Cd-exposed mice. OPs exhibited a strong reductive effect on Cd-induced oxidative stress in the liver.

### 2.7. Effect of OPs on The Hepatic Inflammatory Response (IL-1β, IL-6, TNF-α) in Cd-Exposed Mice

**Figure 5:**
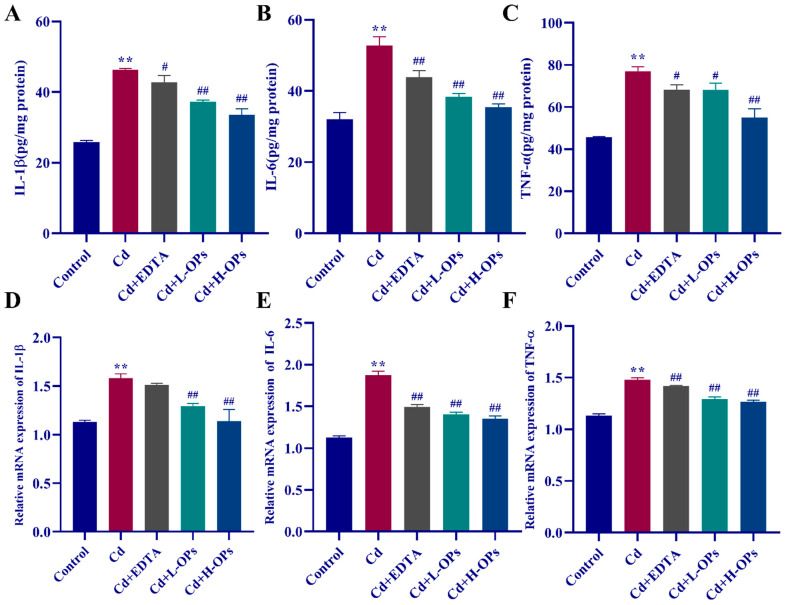
Effect of OPs on inflammatory factor levels and mRNA expression on hepatic in Cd-exposed mice. (**A**) IL-1β level; (**B**) IL-6 level; (**C**) TNF-α level; (**D**–**F**) Hepatic mRNA expression levels of IL-1β, IL-6, and TNF-α in different groups. These data are expressed as the mean ± SEM, *n* = 6 in each group. Compared with the control group, ** *p* < 0.01; compared with the Cd group, ^#^ *p* < 0.05, ^##^ *p* < 0.01.

Interleukin IL-1β and IL-6 are two stimulators of the hepatic synthesis of acute-phase proteins in the inflammatory response to stress and important biological markers of hepatic inflammation [25,26]. TNF-α is an important biological marker in substantial hepatic tissue damage [27]. As shown in Figure 5A–C, the mice in the Cd-exposed group have the highest level of hepatic inflammatory cytokines (IL-1β, IL-6, and TNF-α) (*p* < 0.01). OPs significantly attenuated Cd-induced the high level of hepatic IL-1β, IL-6, and TNF-α (*p* < 0.01 and *p* < 0.05, respectively). The results from quantitative reverse-transcription PCR analysis (qRT-PCR) manifested that OPs inhibited the expression of hepatic IL-1β, IL-6, and TNF-α in Cd-exposed mice (*p* < 0.01). These results revealed that the protection of OPs against Cd was associated with its attenuation of the Cd-induced hepatic inflammation in Cd-exposed mice.

### 2.8. Effect of OPs on The Expression of Hepatic COX-2, MIP-2, NF-κB, and p-ERK in Cd-Exposed Mice 

**Figure 6:**
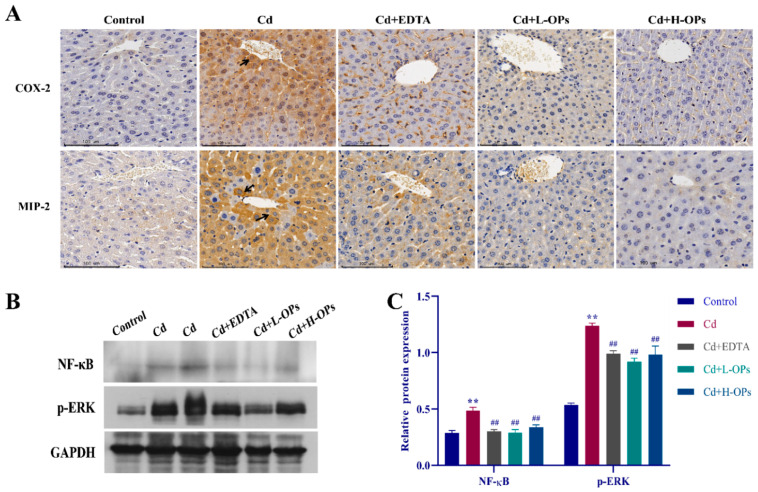
Effect of OPs on the expression of COX-2, MIP-2, NF-κB, and p-ERK in the liver. (**A**) The expression of COX-2 and MIP-2 in the liver by Immunohistochemical (IHC) Staining; (**B**) Western blot analysis of NF-κB and p-ERK proteins, Cd was sampled in 10 µL and 20 µL volumes, respectively; (**C**) Quantitative densitometric analysis of NF-κB and p-ERK proteins. These Data are expressed as the mean ± SEM, *n* = 6 in each group. Compared with the control group, ** *p* < 0.01; compared with the Cd group, ^##^ *p* < 0.01.

COX-2 is a key enzyme in initiating hepatic inflammatory reactions [28]. Meanwhile, macrophage inflammatory protein (MIP)-2 is a potent neutrophil attractant and activator, contributing to the pathogenesis of inflammatory diseases [29]. MIP-2 and COX-2 would be elevated in Cd-induced inflammation [30]. As shown in Figure 6A, enhanced COX-2 and MIP-2 staining were observed around the central vein of hepatocytes in the Cd-exposed group. OPs treatment noticeably reduced the hepatic COX-2 and MIP-2 levels. 

In the process of Cd-induced inflammation, the extracellular signal-regulated kinase (ERK) signal pathway would be activated, and the nuclear factor-κB (NF-κB) subsequently was up-regulated [31]. Western blotting assays illustrated that the expression of NF-κB and p-ERK were highly induced in the Cd-exposed group. However, the OPs treatment effectively dampened the expression of NF-κB and p-ERK (*p* < 0.01; Figure 6B,C). The above results implied that OPs might alleviate hepatic inflammation by inhibiting the expression of inflammatory activators (COX-2 and MIP-2) and related inflammatory pathways (NF-κB and ERK).

### 2.9. Effect of OPs on Hepatic Apoptosis in Cd-Exposed Mice

**Figure 7:**
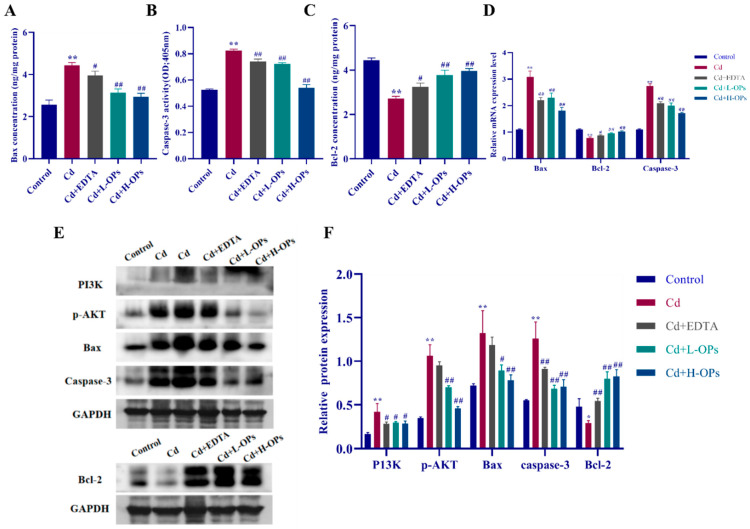
Effect of OPs on apoptotic marker levels and mRNA expression of Cd-induced mice in the liver. (**A**) Bax concentration; (**B**) Caspase-3 activity; (**C**) Bcl-2 concentration; (**D**) Relative mRNA expression levels of Bax, caspase-3 and Bcl-2; (**E**) Western blot analysis of PI3k, p-AKT, Bax, caspase-3 and Bcl-2 protein expression, Cd was sampled in 10 µL and 20 µL volumes, respectively; (**F**) The quantitative densitometric analysis of PI3k, p-AKT, Bax, caspase-3 and Bcl-2. These Data are expressed as the mean ± SEM, *n* = 6 in each group. Compared with the control group, * *p* < 0.05, ** *p* < 0.01; compared with the Cd group, ^#^ *p* < 0.05, ^##^ *p* < 0.01.

Apoptosis-related mitochondrial Bcl2-associated X protein (Bax) and Caspase-3 are two important pro-apoptotic factors. Under the stimulation of oxidative stress caused by Cd, the hepatic Bax increased, then the downstream Caspase-3 was up-regulated, and eventually, apoptosis occurred [32]. Anti-apoptotic Bcl-2 plays a central regulatory role in apoptosis [33]. Accordingly, we examined the effect of OPs on Cd-induced hepatic apoptosis by measuring the levels of pro-apoptotic factors (Bax and caspase-3) and anti-apoptotic factor Bcl-2 in Cd-exposed mice. As shown in Figure 7A–C, Cd significantly decreased the levels of anti-apoptotic Bcl-2 but increased the levels of pro-apoptotic factors (Bax and caspase-3) (*p* < 0.01). On the contrary, OPs treatment significantly increased Bcl-2 while decreasing Bax and caspase-3 levels in Cd-exposed mice (*p* < 0.01). The qRT-PCR results showed that OPs significantly induced the expression of Bcl-2 while suppressing the expression of Bax and caspase-3 in Cd-exposed mice (*p* < 0.01). OPs exhibited a strong anti-apoptotic effect on Cd-induced apoptosis in mice.

Cd can regulate the PI3K/AKT signaling pathway to induce apoptosis [34,35]. Additionally, PI3K/AKT signaling pathway also plays a crucial role in the regulation of inflammatory protein expressions (COX-2 and MIP-2) [36]. Western blotting assays demonstrated that Cd exposure led to the elevation of the expression of PI3K and p-AKT, accompanied by the imbalance of pro-/anti-apoptotic proteins (Bax, caspase-3 and Bcl-2) (*p* < 0.05). By contrast, the OPs treatment inhibited the activation of the PI3K/AKT signaling pathway and restored the balance of pro-/anti-apoptotic proteins in Cd-exposed mice. The results implied that OPs might alleviate hepatic apoptosis by restoring the balance of pro-/anti-apoptotic proteins via inhibiting the PI3K/AKT signaling pathway in Cd-exposed mice.

## 3. Discussion

As one of the main target organs of Cd, acute hepatic injury was observed in Cd-exposed mice. Fortunately, the OPs treatment clearly ameliorated the Cd-induced hepatic injury in this study. In particular, oxidative damage, inflammation, and cell apoptosis, as crucial triggers and contributors to the development of Cd-induced hepatic injury [30,34,35,37], were improved after OPs application in Cd-exposed mice.

Extensive literature indicates that the health benefits of protein hydrolysates may be partly attributed to their rich in free amino acids and peptides [38,39]. Extracts rich in free amino acids can be used in pharmaceutical applications [40]. A recent study found that free amino acids were related to the antioxidant property of protein hydrolysates of mackerel [38]. The protein hydrolysates with higher contents of free amino acids exhibited better antioxidant properties [41] and metal-chelating ability [42]. In the present study, a high level of free amino acids (15.8%) was detected in OPs. According to a previous report, the royal jelly hydrolysates with 8.389% of free amino acids had a stronger antioxidant activity than those of royal jelly with 0.572% of free amino acids [43]. A similar study also found the anchovy sprat hydrolysates with higher contents of free amino acids (16.28–27.53%) exhibited stronger ferrous-chelating activity and radical-scavenging activity compared to those with lower contents of free amino acids (9.05%) [44]. These data indicated that OPs were rich in free amino acids, which may contribute to the potential health benefits of OPs against Cd toxicity.

Serum ALT and AST are leaked from damaged hepatocytes [45,46]. Cd intoxication led to a significant elevation in the levels of ALT and AST [47]. In the present study, significant improvements were observed in the hepatic injury and dysfunction biomarkers (serum AST, ALT, ALP, and LDH) in Cd-exposed mice after the OPs treatment. Compared to the Cd group, OPs significantly decreased hepatic dysfunction biomarkers in a dose-dependent manner. This result is in line with an earlier report, in which oyster protein hydrolysate could reduce hepatic dysfunction biomarkers (serum AST, ALT, and ALP) and inflammatory response in alcoholic liver disease mice [48]. Likewise, Shi, Sun [49] reported that *ganoderma lucidum* peptides have an alleviative effect on D-GalN-induced hepatocellular injury via reversing AST and ALT levels in the liver. Moreover, Mumtaz, Ali [50] found that elevated level of LDH, AST, and ALT in the Cd-exposed batch was improved by ascorbic acid. Early evidence indicates that hepatic injury and cirrhosis usually lead to metabolic disturbances of amino acids [51]. The bioactive properties of protein hydrolysates mainly depend on free amino acids and peptides [38,39,52]. The present study showed that OPs are rich in hydrophobic free amino acids (i.e., Pro) and proline-rich peptides. Among these amino acids, Pro plays a beneficial role in plants under changing environments, including Cd stress [53]. Exogenous Pro could increase antioxidant enzyme activities and confer tolerance to cadmium stress in cultured tobacco cells [22]. Pro has shown tissue-protective effects against D-galactosamine-induced hepatic injury [54]. Dietary Pro could effectively decrease AST and ALT levels of shrimp under NH3 stress [55]. The derivatives of Pro, N-acetyl-seryl-aspartyl-lysyl-proline, were found to attenuate bile duct ligation-induced liver fibrosis by restoring hepatic dysfunction (serum AST and ALT) in mice [56]. Pro and proline-rich proteins are often implicated in stress tolerance in plants [57,58,59]. Salivary proline-rich proteins possess good antioxidant properties [60]. Hypothalamic proline-rich polypeptides were found to protect brain neurons in aluminum neurotoxicosis [61]. These data support the idea that OPs could ameliorate hepatic injury and improve hepatic dysfunction in Cd-exposed mice.

Oxidative stress is often implicated in the induction of multi-organ injury under Cd exposure [62]. Lipid peroxidation is a major consequence of Cd-induced oxidative stress [63]. The consequences of the peroxidative of membrane lipids have been considered in relation to the tissue aspects of liver injury, and these peroxidative reactions play a critical role in the pathogenesis of acute liver necrosis [64]. According to a previous report, the liver, kidneys, and heart were most susceptible to Cd-induced oxidative stress in mice [65]. Some amino acid derivatives, such as N-Acetylcysteine, showed ameliorative effects on cisplatin-induced multiple organ toxicity in rats [66,67,68]. Betulinic acid was found to alleviate the kidney and liver damage induced by Cd [69]. In this study, Cd exposure induced serious hepatic toxicity and oxidative stress, which were significantly improved after the OPs supplement. These data suggest that amelioration of hepatic oxidative injury may be the key to the treatment of Cd toxicity by OPs in mice.

Oxidative stress plays a crucial role in Cd-induced hepatic toxicity [70]. The development of liver injury usually involves the lipid peroxidation of hepatic cell membranes in Cd-exposed mice [71]. According to a recent report, Cd-induced hepatic injury is tightly coupled with enhanced lipid peroxidation (MDA) and the significant depletion of antioxidants (CAT and SOD) [72]. In this study, the OPs supplement clearly reduced the formation of MDA and significantly restored the activity of antioxidant enzymes (SOD, CAT, and GPH-Px) in the liver of the Cd-exposed mice. OPs displayed a strong antioxidant activity, which might also be attributed to their abundance of hydrophobic amino acids. Commonly, protein hydrolysates with higher content of hydrophobic amino acids possess better antioxidant properties due to their more effective interaction with lipid-soluble free radicals and the prevention of lipid peroxidation [17,73,74,75]. Thus, this evidence clearly indicated that OPs possess good antioxidant properties to delay hepatic oxidative injury via retaining hepatic antioxidant enzymes and preventing MDA production in Cd-exposed mice.

Hepatic histopathological damage in Cd-exposed mice is characterized by apoptotic bodies, hemorrhage, lymphocyte accumulation, and inflammatory cell infiltration in liver tissue. Increasing evidence demonstrates that hepatic injury and fibrosis are accompanied by the elevation of the inflammatory response [76]. As well known, TNF-α and IL-6 are two key inflammatory mediators of tissue injury-induced inflammatory response [77]. IL-1β and IL-6 are two stimulators of the hepatic synthesis of acute-phase proteins in the inflammatory response to stress [25,26]. Cd exposure will trigger an acute inflammatory response in mice [78]. The present study showed that hepatic apoptotic cells in Cd-exposed mice were significantly minimized, and histopathological appearance was obviously improved after OPs treatment. In addition, the Cd-triggered inflammatory responses (IL-1β, IL-6, and TNF-α) were significantly inhibited, as expected. This result is consistent with the findings in an earlier report, in which peptides from oyster soft tissue hydrolysates selectively repressed TNF-α, IL-1β, and IL-6 [79]. To go even further, we found that as important activators and regulators of inflammatory responses, MIP-2 [29], COX-2 [28], NF-κB, and the ERK signal pathway [80], were significantly stimulated in Cd-exposed mice. The results are in agreement with a previous study, in which Cd activated the ERK signal pathway, then subsequently up-regulated TNF-α, COX-2, IL-1β, IL-6, and NF-κB in swine [31]. Likewise, Huang, Xia [30] reported that Cd exposure led to an increase in MIP-2 and COX-2. Actually, ROS production could activate MAPK signaling to induce inflammation and skin aging by promoting the phosphorylation of ERK [32]. Peng, Chen [81] also found that the up-regulated ERK phosphorylation in ultraviolet B-exposed mice was significantly inhibited by the application of oyster protein hydrolysates. A recent study found that seahorse protein hydrolysates could significantly inhibit p-ERK levels in ethanol-exposed cells. [82]. In this study, Western blotting assays showed that OPs significantly decreased the levels of p-ERK and NF-κB proteins, as well as the MIP-2 and COX-2 in Cd-exposed mice. Therefore, the ameliorative effect of OPs in Cd-caused liver injury may be related to its anti-inflammation properties via suppressing the production of inflammatory mediators and inhibiting the inflammatory response associated with NF-κB and the ERK signal pathway.

In addition to inflammatory responses, hepatic injury is also accompanied by the development of apoptosis in Cd-exposed mice. Reducing Cd-induced apoptosis is also considered to be one of the feasible ways to prevent Cd-induced hepatic injury [69]. In the process, the NF-κB inflammation pathway indirectly activated the apoptosis-related factors Bcl-2, Bax, and Caspase-3 [31]. The present study revealed that the OPs supplement strongly up-regulated the expression of the anti-apoptotic factor Bcl-2 while significantly down-regulated the expression of the pro-apoptotic factors (caspase-3 and Bax), eventually restoring the balance of pro-/anti-apoptotic proteins in Cd-exposed mice. Moreover, the PI3K/AKT pathway is an important signaling pathway associated with apoptosis [83]. Actually, Cd selectively induces MIP-2 and COX-2 through the activation of the PI3K/AKT [30]. A previous study showed that curcumin alleviated lipopolysaccharide-induced hepatic injury and apoptosis via inhibiting the PI3K/AKT and NF-κB pathways [84]. MiR-130a alleviated neuronal apoptosis and changes in the expression of Bcl-2/Bax and caspase-3 in cerebral infarction rats through the PI3K/AKT signaling pathway [85]. In the present study, we found that the OPs supplement significantly inhibited the expression of PI3K and p-AKT proteins. These results are in agreement with an earlier report, in which Selenomethionine ameliorated Cd-induced hepatocyte apoptosis by suppressing the PI3K/AKT pathway [86]. Therefore, we may conclude that OPs possess the ability to ameliorate hepatocyte apoptosis, possibly by restoring the balance of pro-/anti-apoptotic proteins via suppressing the PI3K/ AKT pathway in Cd-exposed mice.

**Figure 8:**
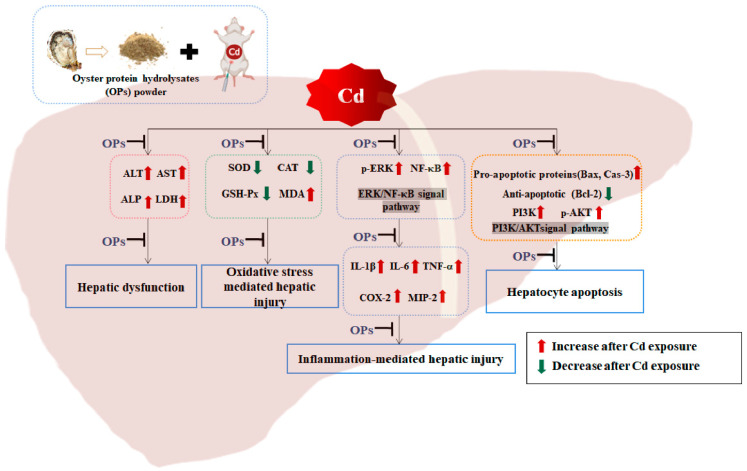
Schematic representation of OPs improved mechanisms of cadmium-induced oxidative stress, inflammation, and apoptosis in mice.

In conclusion, from our study, we found that OPs could effectively ameliorate Cd-induced hepatic injury through their antioxidative and anti-inflammatory properties. In addition, OPs displayed an important role in restoring the balance between pro-apoptotic and anti-apoptotic proteins by suppressing the activation of the PI3K/AKT pathway, contributing to the development of hepatocyte apoptosis in Cd-exposed mice (Figure 8). These results may provide a new insight for a better understanding of the ameliorative function of OPs to Cd toxicity and provide a theoretical basis for the use of OPs to prevent or treat Cd-induced hepatic injury.

## 4. Materials and Methods

### 4.1. Chemical and Materials

Cadmium chloride (CdCl_2_) was purchased from West Long Chemical (Shantou, Guangdong, China). Fresh oysters (*Crassostrea hongkongensis*) were purchased from the local market in Zhanjiang, China. The kits for measurement of ALT (C009-2-1), AST (C010-2-1), ALP (A059-2-2), and LDH (A020-2-2) were offered by Nanjing Jiancheng Bioengineering Institute (Nanjing, China). The kits used to measure the levels of SOD (BC0170), GSH-Px (BC1195), CAT (BC0205), and MDA (BC0025) were purchased from Solarbio Science & Technology (Beijing, China), and the other chemicals were purchased from Sangon Biotech (Shanghai, China) unless specifically noted otherwise.

### 4.2. Animals and Experimental Design

Thirty Specific-Pathogen-Free (SPF) mice (Kunming mice, 25–35 g) were obtained from Changsha Tianqin Biotechnology Co., Ltd. (Changsha, China), and the animals were maintained at the Guangdong Ocean University Animal Centre under light (12 h of light and dark) and temperature (~25 °C). The animals were given a standard laboratory diet and water. The experiment was approved by the Animal Ethics Committee of Guangdong Ocean University (No.: GDOU-LAE-2020-009). The animals were randomly divided into 5 groups (*n* = 6). Control group: Intraperitoneal injection of 0.9% NaCl (saline) once daily. Cd-exposed group: the mice were injected intraperitoneally with CdCl_2_ 5 mg/kg daily [87]. EDTA-treated group (positive control): the mice were injected with CdCl_2_ (5 mg/kg) intraperitoneally after 1 h of oral administration with EDTA (100 mg/kg) daily. The low dose of OPs(L-OPs)-treated group: the mice were injected with CdCl_2_ (5 mg/kg) intraperitoneally after 1 h of oral administration with OPs (100 mg/kg) daily. High dose of OPs (H-OPs)-treated group: the mice were injected with CdCl_2_ (5 mg/kg) intraperitoneally after 1 h of oral administration with OPs (300 mg/kg) daily. It has been shown that ethylenediaminetetraacetic acid (EDTA) can alleviate cadmium toxicity by enhancing antioxidant enzyme activity and inhibiting inflammatory responses [88]. Therefore, it can be used as a positive control. The doses of EDTA and OPs were determined based on previous studies [89,90]. After 7 days, the mice were executed by cervical dislocation, and the liver was stored at −80 °C for further analysis.

### 4.3. Preparation of OPs

OPs were prepared by enzymatic hydrolysis from the oyster (*Crassostrea hongkongensis*) meat as described previously [81,90]. Hong Kong oyster meat (~3 kg) was homogenized in distilled water (1:3 (*w*/*w*) at 8000 rpm for 5 min. Homogenized oysters were hydrolysed using neutral protease (2 × 10^5^ U/g, Pangbo Biotech, Nanning, China) at a protease/substrate ratio of 2.0% (*w*/*v*) (pH 7.0). The neutral protease was incubated for 4 h at 50 °C and then inactivated at 100 °C for 15 min. The hydrolysate was centrifuged at 15,000 rpm for 20 min at 4 °C to obtain the supernatant. The supernatant was collected and ultrafiltered using a membrane bioreactor system with a molecular mass cut-off value of 3 kDa to collect the fractions (<3 kDa). The samples were collected and freeze-dried for further analysis.

### 4.4. Peptide Sequence Analysis Based on LC-MS/MS

The peptide sequence analysis used an Easy-nLC 1200 system coupled to a Q-Exactive quadrupole-Orbitrap mass spectrometry (Thermo Fisher Scientific, San Jose, CA, USA). One μL of the samples was injected with an autosampler into an Acclaim Pep Map RPLC C18 column (150 μm i.d. × 150 mm, C18, particle size: 1.9 μm, pore size: 100 Å, Dr. Maisch GmbH, Darmstadt, Hessen, Germany) with mobile phase A: 0.1% formic acid in water; B: 0.1% formic acid in the water, 80% acetonitrile. The flow rate was 600 nL/min, and the LC linear gradient ranged from 4% to 40% for 55 minutes and 10 minutes at 95% mobile phase B. Finally, the molecular mass, sequence, peak area (with respect to base peak), and relative peak area (peak area/total peak area) of the peptides were identified and calculated as previously described [81,91]. The conditions of the mass spectrometer were as follows: Resolution: 70,000, AGC target: 3e6, NCE/stepped NCE: 28. The samples were analyzed with a full-scan MS mode in the range of 100–1500 *m*/*z* to obtain the total ion chromatogram. The raw MS files were analyzed and searched against the target protein database based on the species of the samples using Byonic. 

### 4.5. LC-MS/MS Analysis of Free Amino Acids 

The amino acid composition and content of the OPs were measured, as previously described, with little modification [92]. The OPs samples were accurately weighed to 50 mg and mixed with 600 μL of a water–methanol solution (1:1, *v*/*v*) with 10% formic acid in a 2 mL tube. Then, 100 mg of glass beads were added to the mixed samples and vortexed for 30 s. The samples were transferred to a high-throughput tissue grinding machine (MB-96, Meibi, Jiaxing, Zhejiang, China) and vibrated at 60 Hz for 2 min. The tube was centrifuged at 12,000 rpm for 5 min at 4 °C. Ten μL of supernatant was transferred to a new tube containing 490 μL of the water–methanol solution (1:1, *v*/*v*) with 10% formic acid and then vortexed for 30 s. Then, 100 μL of the diluted samples were mixed with 100 μL of 100 μg/L double isotope internal standard (Trp-d3, D87103, Medical Isotopes, USA) and vortexed for 30 s. The mixed samples were filtered through a 0.22 μm hydrophilic PTFE filter and transferred into a labeled vial, and subsequently analyzed via LC–MS/MS.

Five μL of the samples were injected into an ACQUITY UPLC BEH C18 column (2.1 × 100 mm,1.7 μm, Waters, Milford, MD, USA) with mobile phase A: 10% water -methanol solution with 0.1% formic acid; B: 50% water-methanol solution with 0.1% formic acid. The flow rate: 300 μL/min in 8.5 min, then kept 300–400 μL/min for 4 min. The gradient elution programs: 0~6.5 min, 10~30% B; 6.5~7 min, 30~100% B; 7~8 min, 100% B; 8~8.5 min, 100~10% B; 8.5~12.5 min, 10% B.

Mass spectrometric analysis was performed using an AB SCIEX AB4000 Mass Spectrometer (AB SCIEX, Framingham, MD, USA) equipped with an electrospray ionization (ESI) source using the following parameters: capillary voltage: 5500 V, temperature of the turbo heaters: 500 °C, nebulizer gas (GS1): 50 psi, auxiliary gas (GS2): 50 psi, and curtain gas (CUR): 30 psi, Collision Gas: 6 psi. All of the amino acids were detected in the multiple reaction monitoring mode (MRM).

### 4.6. Analysis of Amino Acid Composition

According to the previous method with a slight modification [81]. Approximately 30 mg of the sample and 10 mL of 6 mol/L HCl were added to a hydrolysis tube containing phenol. After the tube was vacuumed, the mixture was washed with nitrogen and hydrolyzed at 110 °C for 22 h. After cooling to room temperature, the filtrate is filtered and spun dry under reduced pressure in a centrifuge tube. The 0.02 mol/L HCl solution was added to a dried centrifuge tube and dissolved, and the solution was transferred to the upper sample bottle and determined using an amino acid analyzer (L-8900, Hitachi, Tokyo, Japan). Then, the contents of amino acids in the sample could be determined according to the peak area in comparison with the standard. The content of tryptophan in OP was determined after hydrolysis with 6 mol/L of NaOH instead of HCl.

### 4.7. Histopathology Examination

The liver samples were fixed in 4% paraformaldehyde for 24 hours and embedded in paraffin. The embedded liver tissue was sectioned into 5 µm sections and fixed on slides, stained with hematoxylin and eosin (H&E) and observed under a BX 53 Olympus microscope according to the method described [93].

### 4.8. Analysis of Liver Function

The blood samples were collected as previously described [94,95]. Blood samples were gained by removing the eyeballs of mice. Blood was then centrifuged at 4000 rpm for 30 min at 4 °C. The samples were incubated in an electro-thermostatic water bath at 37 °C for 30 min. The serum was collected and subjected to the examination of the activities of alanine transaminase (ALT), aspartate aminotransferase (AST) activities, the activities of lactate dehydrogenase (LDH), and alkaline phosphatase (ALP) with respective commercial kits. The determination of AST, ALT, LDH, and ALP was performed by the instruction of the kits (Nanjing Jianchen Bioengineering Institute, Nanjing, China).

### 4.9. Measurement of MDA, SOD, CAT and GSH-Px Activities

The changes in hepatic oxidative stress were monitored as previously described [65]. The liver homogenate was centrifuged to obtain the supernatant at 3500 rpm for 10 min at 4 °C, and the superoxide dismutase (SOD), catalase (CAT), glutathione peroxidase (GSH-Px) activity, and malondialdehyde (MDA) levels were measured according to the manufacturer’s instructions.

### 4.10. Measurement of IL-1β, IL-6 and TNF-α in Hepatic Tissue by ELISA Kits

The concentrations of IL-1β (MM-0905M1), IL-6 (MM-1011M1), and TNF-α (MM-0679M1) were measured using an ELISA kit (MeiMian, Yancheng, Jiangsu, China) according to the manufacturer’s instructions.

### 4.11. Immunohistochemistry Analysis

MIP-2 and COX-2 expressions in hepatic tissue were evaluated by immunohistochemistry staining as previously described [96]. The prepared hepatic sections were recovered, and the endogenous peroxidase in tissues was inactivated with 0.1% hydrogen peroxide containing methanol for 15 min. Then, the sections were incubated with a rabbit polyclonal MIP-2 and COX-2 antibody at 4 °C overnight. Subsequently, the sections were washed with PBS and incubated with rabbit anti-mouse (1:1000) secondary antibody at room temperature for 30 min. The sections were rinsed with PBS 3 times and stained with diaminobenzidine (DAB). Additionally, they were evaluated under an optical microscope (Olympus Optical Co., Ltd., Tokyo, Japan).

### 4.12. Measurement of Bax, Caspase-3 and Bcl-2 in Hepatic Tissue

The levels of pro-apoptotic-related protein Bax (MM-1143H2) and anti-apoptotic-related protein Bcl-2 (MM-0306M2) were performed using an ELISA kit (MeiMian, Yancheng, Jiangsu, China) according to the manufacturer’s instructions. Caspase-3 activity was measured using a kit (C1115) purchased from Beyotime Biotechnology (Shanghai, China).

### 4.13. Quantitative Reverse-Transcription PCR (qRT-PCR) Analyses

The total RNA from each sample was isolated using the Trizol reagent (Sango Shanghai, China), and the first strand cDNA was synthesized using the StarScript II First-strand cDNA Synthesis Mix With gDNA Remover (Genstar) according to the manufacturer’s instructions. Quantitative reverse-transcription PCR (qRT-PCR) was conducted to determine the mRNA levels of the IL-1β, IL-6, TNF-α, Bax, Caspase-3, and Bcl-2 (the primer sequences are shown in Table 4), the GAPDH gene was used as an internal control [8,30,97]. Real-time PCR reactions were performed on a CFX Real-time system (CFX96, Bio-Rad, Hercules, CA, USA). All of the samples were analyzed in triplicate, and the 2^−ΔΔCt^ method was used to analyze gene expression levels.

### 4.14. Western Blotting Analyses

The hepatic tissues were lysed with RIPA lysis buffer (Servicebio technology, Wuhan, China), supplemented with protease inhibitor (Servicebio), and homogenized with an ultrasonic processor. According to the manufacturer’s instructions, the total protein of the liver tissue was extracted with a commercial kit (Servicebio technology, Wuhan, China). Then, the concentration of the protein was measured with a BCA kit (Beyotime technology, Shanghai, China). Then, the proteins were transferred to a polyvinylidene fluoride (PVDF) membrane, followed by blocking with 5% skim milk (0.5% TBST) and sealed for 1 h. Additionally, then, PDVF membranes were incubated with primary antibodies against NF-κB (1:1000), p-ERK (1:1000), PI3K (1:1000), p-AKT (1:1000), Caspase-3 (1:1000), Bcl-2 (1:1000), Bax (1:1000), and GAPDH (1:3000) were incubated overnight at 4 °C. They were washed with TBST at room temperature on a decolorizing shaker three times. After washing, PVDF membranes were incubated with secondary antibodies (1:3000) at room temperature for 2 h. The antibodies were purchased from Proteintech Group, USA. Finally, they were developed and fixed with developing and fixing reagents, and the Alpha software processing system analyzes the optical density values of the target band.

### 4.15. Statistical Analysis

The data are expressed as the mean ± SE. Data analyses were carried out using JMP Pro 13. The data were analyzed using general descriptive statistics. One-way analysis of variance (ANOVA) at 95%. *p* < 0.05 was considered statistically significant.

## Figures and Tables

**Table 1:** Main peptide sequences of OPs.

Rank	Peptide Sequence	Length	Molecular Mass (Da)	Observed Mass (*m*/*z*)	Peak Area	Relative Peak Area (%)	Scores
1	GEPGPEGPAGPIGPR	15	1387.697	694.350	18,567,000	0.15	614.6
2	YEETRGVLK	9	1094.584	547.795	9,895,900	0.08	494.2
3	GPTGPVGPL	9	794.441	794.437	3,857,400	0.03	477.2
4	GPSGEPGPE	9	826.358	826.354	12,054,000	0.10	468.7
5	DIERPTYT	8	994.484	497.745	18,359,000	0.15	461.4
6	ENPVPVPS	8	838.431	838.427	6,999,200	0.06	453.3
7	TEAPLNPK	8	869.473	869.471	9,380,400	0.08	451.1
8	TPEEFIPR	8	988.510	494.758	12,323,000	0.10	437.8
9	AGFAGDDAPR	10	976.448	488.727	171,200,000	1.39	430.9
10	TPTYGDL	7	766.362	766.359	46,716,000	0.38	428.1
11	PDVPAGDVDKGK	12	1197.611	399.874	14,901,000	0.12	425.1
12	GPIGGPL	7	610.356	610.354	5,358,100	0.04	423.7
13	SPVGVGA	7	586.320	586.318	13,332,000	0.11	411.4
14	YTPVAYPV	8	586.320	586.318	4,007,500	0.03	388.5
15	LTPSGLPY	8	647.342	647.342	14,591,000	0.12	386.7
16	STPFEGF	7	571.308	571.308	13,332,000	0.11	384.4
17	VSDTVVEPYN	10	550.250	550.250	27,505,000	0.22	383.3
18	DIERPTYTN	9	909.420	909.420	8,461,100	0.07	383.2
19	QGETGDRGPFG	11	879.456	440.231	34,716,000	0.28	383.0
20	PRPPTQVGGS	10	995.525	498.266	38,429,000	0.31	382.8

**Table 2:** Composition and content of amino acids in OPs.

Amino Acids	Contents (g/100 g)	Amino Acids	Contents (g/100 g)
Alanine (Ala) ^#^	2.63	Leucine (Leu) *^#^	2.02
Cystine (Cys)	0.40	Methionine (Met) *^#^	0.65
Aspartic acid (Asp)	3.36	Proline (Pro) ^#^	2.61
Glutamic acid (Glu)	4.33	Arginine (Arg)	1.71
Phenylalanine (Phe) *^#^	0.72	Serine (Ser)	1.83
Glycine (Gly)	2.92	Threonine (Thr) *	1.69
Histidine (His)	0.50	Valine (Val) *^#^	1.81
Isoleucine (Ile) *^#^	1.40	Tyrosine (Tyr)	1.62
Lysine (Lys) *	2.61	Tryptophan (Trp) *^#^	0.92
Total amino acids	33.73		
Essential amino acid	11.82		
Hydrophobic amino acids	12.76		

Note: * Essential amino acids. ^#^ Hydrophobic amino acids.

**Table 3:** Contents of free amino acids in OPs.

Free Amino Acids	Contents (g/100 g)	Free Amino Acids	Contents (g/100 g)
Ala ^#^	0.59	Met *^#^	0.36
Cys	ND	Asn	0.26
Asp	0.37	Pro ^#^	2.49
Glu	2.12	Gln	0.85
Phe *^#^	0.18	Arg	0.53
Gly	1.38	Ser	0.29
His	0.08	Thr *	0.84
Ile *^#^	0.59	Val *^#^	0.91
Lys *	1.43	Trp *^#^	0.52
Leu *^#^	0.50	Tyr	1.51
Total free amino acids	15.80		
Essential free amino acid	5.33		
Hydrophobic free amino acids	6.14		

Note: * Essential free amino acids. ^#^ Hydrophobic free amino acids; ND: not detected.

**Table 4:** Primer sequences for qRT-PCR analyses.

Gene Name	Forward Primer (5′–3′)	Reverse Primer (5′–3′)
IL-1β	GACTTCACCATGGAACCCGT	GGAGACTGCCCATTCTCGAC
IL-6	GGCCCTTGCTTTCTCTTCG	ATAATAAAGTTTTGATTATGT
TNF-α	AGCCCTGGTATGAGCCCATGTA	CCGGACTCCGTGATGTCTAAGT
Bax	CTGAGCTGACCTTGGAGC	GACTCCAGCCACAAGAGATG
Caspase-3	GAGCTTGGAACGGTACGCTA	CCGTACCAGAGCGAGATGAC
Bcl-2	GACAGAAGATCATGCCGTCC	GGTACCAATGGCACTTCAAG
GAPDH	TCACCACCATGGAGAAGGC	GCTAAGCAGTTGGTGGTGCA

## Data Availability

The data presented in this study are available on request from the corresponding author.
